# Cybersecurity vulnerability analysis of medical devices purchased by national health services

**DOI:** 10.1038/s41598-023-45927-1

**Published:** 2023-11-09

**Authors:** Lorenzo Bracciale, Pierpaolo Loreti, Giuseppe Bianchi

**Affiliations:** 1 Department of Electronic Engineering, University of Rome Tor Vergata, Rome, Italy; 2 CNIT, National Inter-University Consortium for Telecommunication, Parma, Italy

**Keywords:** Electrical and electronic engineering, Public health, Information technology

## Abstract

The growing integration of software within medical devices introduces the potential for cybersecurity threats. How significant is this risk, and to what extent are citizens currently exposed? In this study, we adopt a new data-gathering methodology using datasets provided in Open Contracting Data Standard (OCDS). This allowed us to perform an extensive analysis across over 36 countries within a 12-year range, searching 92 million public administration purchase records for potentially vulnerable medical devices. The findings reveal a concerning landscape wherein numerous medical devices purchased by national health services possessed or still possess 661 distinct vulnerabilities—more than half of which are deemed critical or high-severity. These vulnerabilities enable relatively simple attacks to impact data confidentiality, integrity, and accessibility severely. Even if patches were applied immediately upon discovery, these vulnerabilities would still result in roughly 3.2 years of system exposure from the time a device is purchased until a software vulnerability is announced, with all classes of devices affected, including high-risk IIB and III devices which accounts for 74% of instances. While a full analysis requires interactivity, this noninvasive methodology enables a large-scale study, emphasizing the need to move faster from the safety to the security of medical devices.

## Introduction

According to the World Health Organization (WHO), there are more than 2 million different types of medical devices^1^.

Medical devices (MDs) are devices that are intended to maintain or improve health, treat medical conditions or diseases, or facilitate the diagnosis or monitoring of medical conditions. These devices comprise a broad spectrum of constantly evolving technologies, with more and more devices involving a software part. For instance, Magnetic Resonance Imaging (MRI) machines use software for signal processing and data visualization. Infusion pumps, which account for 38% of a hospital’s Internet of Things (IoT) footprint^2^, have firmware for control and management. Insulin pumps may use wireless connections to show medical parameters and allow the regulation of drug dosage^3^. The dark side of the story is that all software is potentially vulnerable. Indeed, some MRI machines expose sensitive information^4^; 3 of 4 infusion pumps allow for leakage of sensitive information or allow unauthorized access^5^; some insulin pumps allow even remote attackers to change pump settings and control insulin delivery^6^, with potentially fatal outcomes.

Are medical devices in our homes or hospitals secure against these types of cyber threats? Answering this question is extremely complicated. It depends on the ability of the asset manager (the product owner or a hospital IT manager) not only to patch a device as soon as a new vulnerability comes out, but also to be *aware* that a relevant vulnerability exists and is relevant to their assets. Indeed, security updating of Internet of Things devices is notoriously expensive and far from successful in practice^7^.

Specifically, when there is the disclosure of a new flaw in the software of a medical device, like for any other kind of software, we have the publication of a Common Vulnerabilities and Exposures (CVE), a unique reference for a specific vulnerability, which is published by the US MITRE Corporation and used worldwide by security researchers. To date, there are 211,890 different CVEs, with more than 2500 new CVEs added per month. Clearly, not all such vulnerabilities are of interest to medical purposes. A shortlist of CVEs affecting medical devices is made available by the US Cybersecurity and Infrastructure Security Agency (CISA), through Cybersecurity Alerts and Advisories. Each alert has a specific code, such as ICSMA-XX-YYY-ZZ, and all information is made available on the cisa.org website.

Though the list may be shorter, not every security notice will pertain to a specific healthcare facility. For instance, a facility might not have ever purchased a particular piece of medical equipment. As such, it’s essential to enhance the relevance of cybersecurity alerts to raise awareness. One way to do this is by crafting customized messages related to the actual assets managed by a particular organization. To accomplish this, access to asset lists from all medical facilities is needed—something that typically isn’t shared beyond the facility and primarily serves other purposes, such as inventory management.

In our study, we managed to obtain asset lists for 1241 healthcare facilities by examining 92 million public administration purchases spanning 36 countries over a decade. This information was made available as Open Data via the Open Contracting Data Standard (OCDS) for transparency purposes.

We sifted through this data for medical device purchases and correlated it with cybersecurity information relating to vulnerabilities, weaknesses, and security alerts for MDs. The result is an Open Source Intelligence (OSINT) tool that can provide targeted information on assets to help healthcare managers improve their cybersecurity awareness. Furthermore, this tool enables an analysis of potential vulnerabilities in deployed medical devices, identification of common weaknesses, and evaluation of National Health Systems’ exposure to cyber threats. By processing the data, we generated a detailed map of potentially vulnerable devices. Such high-level analysis can greatly narrow down the field for more specific (and costly) in-depth evaluations on medical device security, optimizing risk assessment’s cost/benefit ratio.

Additionally, we associated the risk class of MDs according to Medical Device Regulation (Regulation (EU) 2017/745) with vulnerability severity determined by the Common Vulnerability Scoring System (CVSS). This offers valuable insight into current National Health Systems risks.

The transition from ensuring the safety of medical devices to also considering their security is gaining momentum rapidly.

Our analysis aligns with the recent US Consolidated Appropriations Act, signed into law on December 29, 2022, which raises the bar on medical device security risk management^8^. Specifically, section 524B explicitly requires “cyber devices” to monitor and address postmarket cybersecurity vulnerabilities in a reasonable time and provide the Secretary with a software bill of materials, including commercial, open-source, and off-the-shelf software components.

## Results

### The analysis

We analyze 92M purchase orders issued by the public administration of 36 countries from 2010 up to today. Such datasets have been made available for the purpose of transparency by many different countries but are rarely used for research. Through data mining techniques, we searched for purchases of medical devices with known vulnerabilities i.e., where CISA emits a cybersecurity alert. Our analysis generates a comprehensive timeline of when healthcare facilities acquired potentially compromised devices. This does not necessarily mean that all such devices are still vulnerable *now*. Indeed, most manufacturers voluntarily recall medical devices that could create health risks^9^, but this procedure may have problems attributable to the chain of notifications between manufacturers, healthcare providers and patients that is not always effective^10^.

In general, the analysis presented in this work (i) can help conduct more cost-effective cybersecurity audit by identifying only a precise subset of devices to be verified; (ii) can be used as an awareness tool already today and without the acquisition of further data; (iii) provides insight into the critical security issues of medical devices in use. In what follows, we indicate as a *match* when we can reasonably attribute a purchase to a potentially vulnerable device. This does not mean that the purchase is about one single device, but can also imply the purchase of spare parts or consumables that indirectly provide us with information on the presence of the device in a certain health facility.

**Figure 1:**
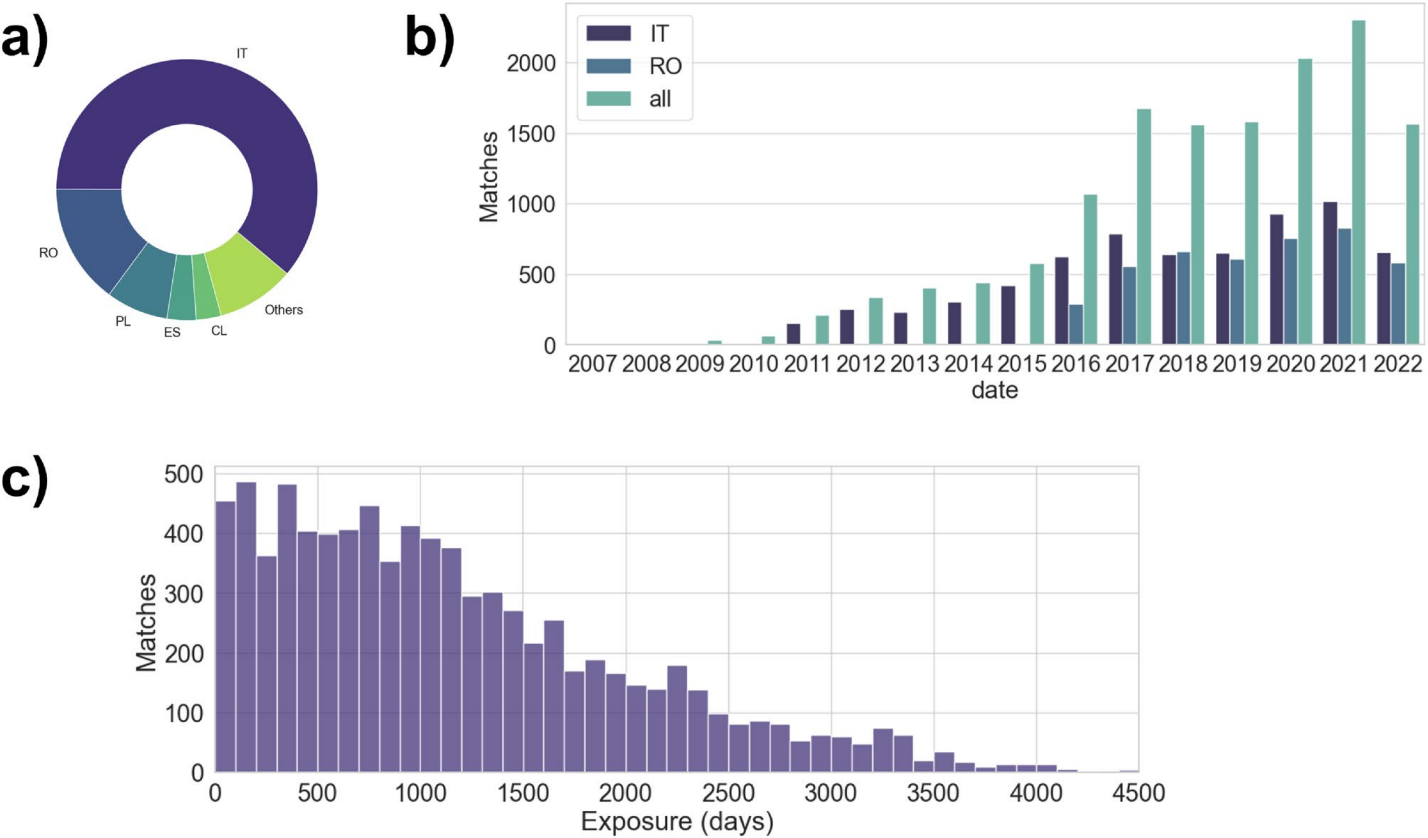
Geographical (**a**) and temporal (**b**) distribution of the analyzed data. Statistics of the time between the purchase of a MD and the release of a CVE (Exposure times) (**c**).

Figure 1a shows the distribution of the matches per country.

From the analysis, we found 14,478 purchases which can be attributed to 202 different types of medical devices bought by 1241 health facilities and having 150 different known vulnerabilities.

Italy accounts for more than half of the cases (6666 matches) since the Italian National Anti-corruption Authority has been collecting from 2011 all the information about public contracting with a high level of precision. Romania comes in second with 4311 cases, as data collection only commenced in 2020. This can be observed in Fig. 1b, which displays the number of purchases involving vulnerable devices per year. Poland ranks third with 1104 instances, followed closely by Spain and Chile with 510 and 412 cases, respectively. Italy and Romania, both of which possess extensive datasets, also implement Universal Healthcare Systems, thereby ensuring that all individuals can access a comprehensive spectrum of high-quality health services as needed without incurring financial burdens. This means that public administration frequently purchases medical devices.

The degree of generalization of the analysis clearly depends on the characteristics of the countries that contribute most to the dataset. We note, however, that most of the manufacturing companies involved are multinationals, which makes the analysis quite indicative of a general trend and scenario.

It is also important to note that countries not included in this analysis aren’t exempt from potential vulnerabilities in their medical equipment; they simply can’t be monitored using this system. Upon examining the data, we can discern a general upward trend, peaking in 2020 due to the pandemic and Romania’s initiation of data collection.

### Exposure

Figure 1c shows how much time passes between the purchase of a medical device and the discovery of a vulnerability for that device. The figure thus provides a clear picture of how exposed health systems are to possible cyber threats affecting MDs.

Even when we assume that all devices are updated with fixes for new vulnerabilities on the day they emerge, there remains an average window of 3.2 years in which these medical devices could potentially expose patients to risks due to existing vulnerabilities.

Our analysis specifically focuses on instances where purchase orders occur before vulnerability discoveries. We do this under the conservative assumption that products ordered after a vulnerability is detected come pre-installed with appropriate patches provided by the manufacturer or reseller.

### Severity

**Figure 2:**
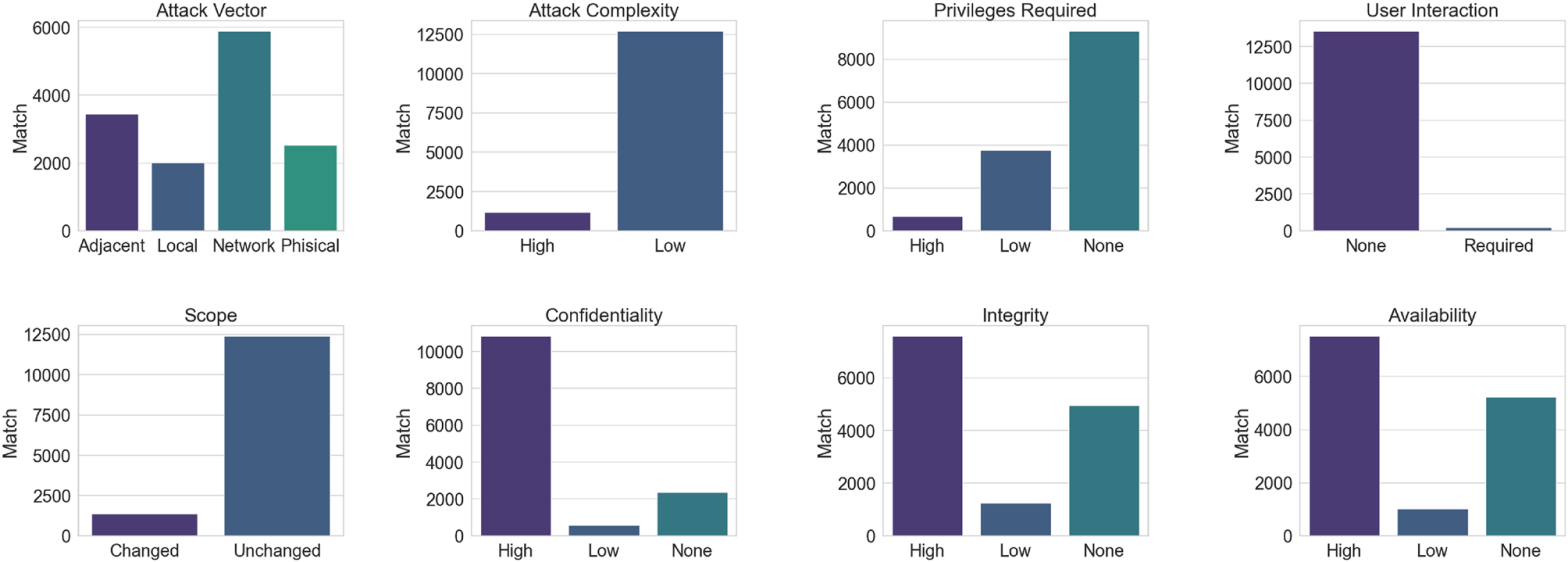
CVSS Base Score Metrics of matched vulnerabilities.

How grave are these security vulnerabilities? What’s the ease of exploitation for potential attackers, and do they pose a risk to life? To address these concerns, we’ve correlated vulnerability data with the CVSS score. The Common Vulnerability Scoring System (CVSS), proposed by the National Institute of Standards and Technology (NIST), serves as an open industry benchmark for gauging the severity of computer system security vulnerabilities. As illustrated in Fig. 2, most discovered vulnerabilities can be exploited remotely (with the network as an attack vector) and exhibit a low attack complexity. A significant majority necessitate no user interaction, and many demand either no or low privileges. While most vulnerabilities affect only resources controlled by the same security authority (unchanged scope), they typically result in a total compromise of confidentiality, integrity, and availability.

**Figure 3:**
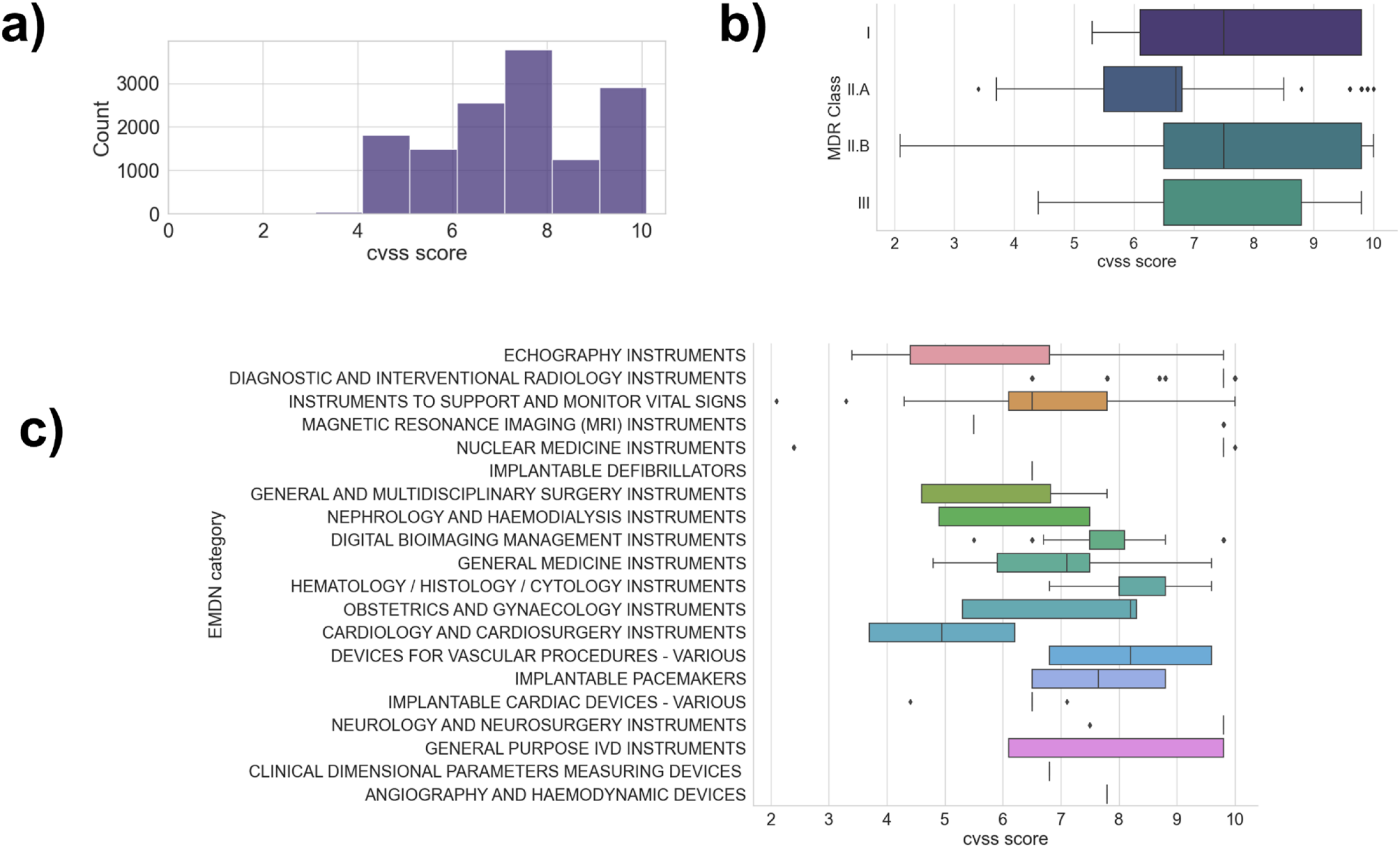
Analysis of CVSS score according to the number of matches (**a**), to the device class (**b**) and to the device EMDN category (**c**).

Figure 3a displays the distribution of CVSS Base scores, representing various vulnerabilities’ severity. These scores range from 0 to 10, with 10 being the most severe. As illustrated in Fig. 3a, a mere 0.4% of the identified vulnerabilities are considered low (CVSS < 4.0), while 42% are classified as medium (4.0 $\le$ CVSS $\le$ 6.9), 36% as high (7.0 $\le$ CVSS $\le$ 8.9), and a significant 20% are labeled critical (CVSS $\ge$ 9.0). This percentage is about two times higher than the average critical vulnerabilities, which are just 9.8% of the total. Figure 2 shows indeed the technical reasons behind such scores: too easy-to-exploit vulnerabilities that result in a severe impact on the confidentiality, integrity, and availability of medical devices.

### Risk

It’s crucial to understand that CVSS quantifies severity rather than risk. In essence, it evaluates the likelihood of a device malfunctioning but doesn’t assess the probability or severity of physical damage. As a result, it’s not directly applicable to risk measurement in accordance with ISO 14971^11^. However, CVSS is undoubtedly instrumental in evaluating risks.

To offer more precise data for risk analysis, we’ve categorized the devices under examination based on their respective risk classes. According to Article 51 of the Medical Device Regulation (MDR, EU 2017/745), medical devices are divided into four classes depending on their purpose and inherent risks: class I, class IIa, class IIb, and class III. Class I devices pose the lowest risk to patient safety; class IIa and class IIb represent medium and medium/high-risk devices (e.g., those used to administer or remove medicinal products); and class III encompasses devices with the highest risks, such as implantable devices or pacemakers^12^.

From our analysis, class II.B accounts for 73% of the matches (10,121), followed by class II.A devices (2667 matches) and class III devices (174 matches). Class I has only 81 results and 817 matches are not catalogable. As we can see from the data plotted in Fig. 3b, vulnerabilities with high CVSS scores affect all classes across the board. Notably, Class II.B devices have an impressive average CVSS score of 7.5.

To better understand the types of devices being discussed, we turned to the European Medical Device Nomenclature (EMDN)^13^. Figure 3c illustrates our analysis, in which we combined various devices up to level 3 of the EMDN hierarchy. Interestingly, the results demonstrate a minimal correlation between operational fields and vulnerability severity; vulnerable devices range from ultrasound to cardiology equipment.

### Weaknesses

What causes these vulnerabilities? To shed some light on this, we connected our dataset to the Common Weakness Enumeration—a comprehensive classification system containing over 600 types of hardware and software weaknesses. A weakness is essentially a flaw that gives rise to a specific vulnerability, such as buffer overflows or cross-site scripting.

Our analysis identified the top 10 weaknesses, which account for up to 59% of all cases. The most common issue is the use of hard-coded credentials (CWE-798), accounting for 9% of cases, followed closely by authorization problems (CWE-200).

**Figure 4:**
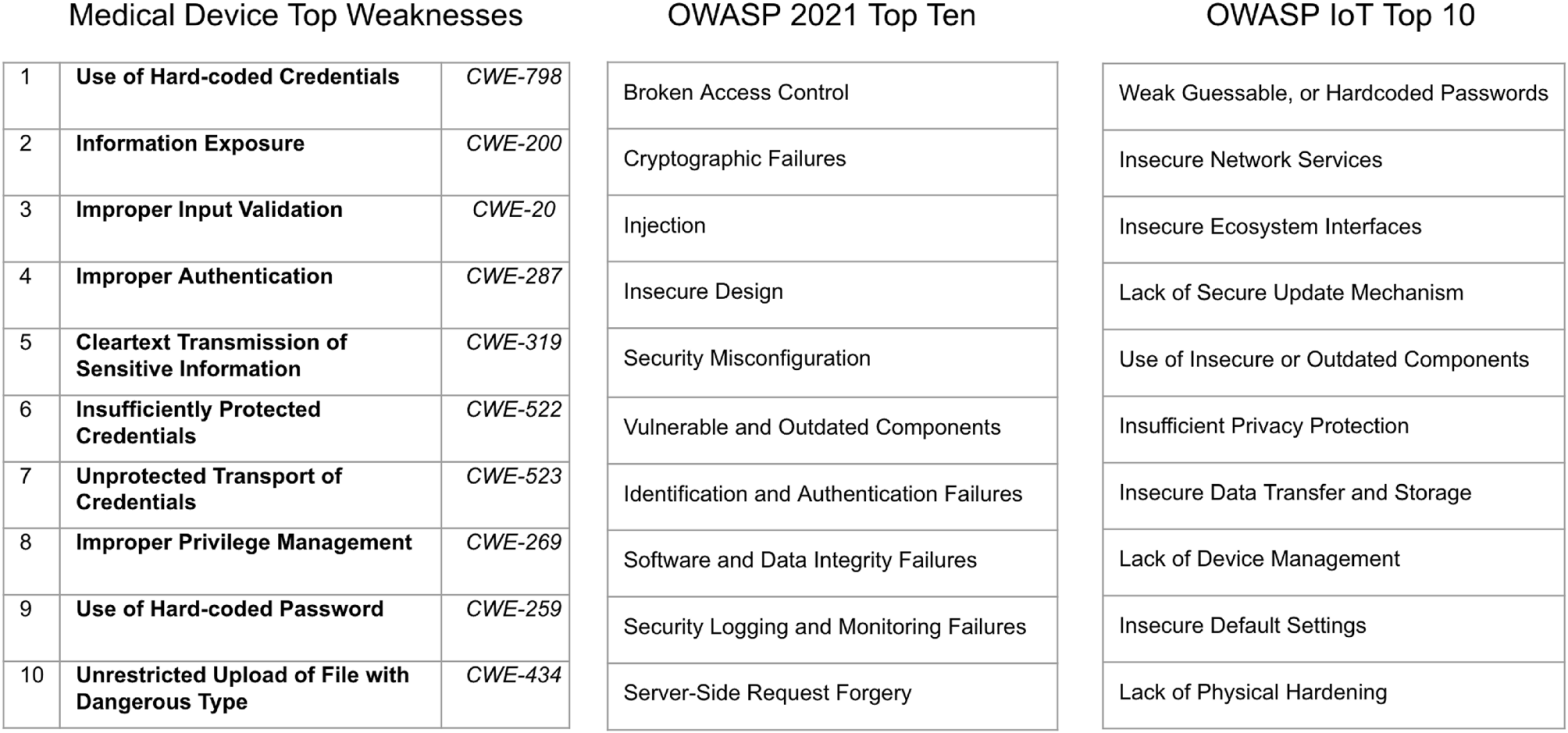
Top medical devices weaknesses vs most dangerous weaknesses (2021) vs most dangerous weaknesses IoT weaknesses (2018).

In Fig. 4, we compare the rankings of medical devices’ weaknesses to the top 10 weaknesses in the web world for 2021 (OWASP-10 in the second column)^14^ and the top 10 weaknesses in the IoT world (OWASP IoT 10 displayed in the third column with the most recent version from 2018). The weaknesses found in medical devices differ from those in the web world. For example, CVE-2021-33882 reports a total lack of authentication in a medical device^15^. In contrast, the web world frequently experiences authentication issues (#7 in Fig. 4), but total absences are rare. Medical devices and IoT devices share several similarities in their weaknesses, such as the problem of hardcoded credentials or the transmission of data using clear text. This means that medical devices, sometimes belonging to MDR risk classes II.B or III as shown in Fig. 3b, may have similar vulnerabilities to consumer devices such as smart bulbs or IoT smart speakers.

## Discussion

In this study, we presented a new methodology that we used to perform an analysis of the status of medical devices from a cybersecurity perspective. The analysis raises the following points for discussion:

- **Designed for safety, not for security**: The analysis shows how medical device vulnerabilities are generally more severe than those concerning non-medical products. Indeed, critical severity affects 20% of cases, which is well above the average critical vulnerability that currently stands at 9.8%. A good interpretation of the result is given by Douglas McKee and Philippe Laulheret’s technical analysis of the software of an infusion pump^15^. From the report, it is clear how a well-engineered product was designed with safety in mind and not for security. In fact, while various precautions have been taken, for example, against possible memory faults, for the communication part everything has been supposed to be trusted. Given the growth of offer of “cyber devices” and their purchases, this approach needs a radical change.
- **NHS are exposed for long times**: The analysis shows how vulnerabilities become public after on average 3.2 years after purchasing a medical device, leaving a remarkable window where devices are exposed to attacks. Indeed, many medical equipment (e.g. CT or MRI devices) have in general a much longer lifespan than ordinary software which also introduces the problem of managing the “queue” of devices not designed for security. Regarding the current status of these devices, although we have not been able to verify through open data their actual troubleshooting, we know anecdotally that many have undergone recall procedures. Recall is a complex procedure that requires notifications from manufacturers to healthcare providers to patients. Such notification chain is fragile and can be very slow due, for example, to difficulties in individuating people actually responsible for pulling products from hospital inventories^10^. What is certain is that the patching procedure is complicated and time-consuming as it is multi-stage^7^. If the software vendor issues a patch, the device manufacturer must conduct an engineering study prior to verification and certification. Testing in the target context and an effective study on patient safety, workflow, scheduling, and patient care are necessary after the product is released to the healthcare provider. Finally, the patch must be distributed and installed on all the devices^16^. This process results in long times in which 0-day attacks would have time to be prepared and carried out.
- **Defense or offense?**: The kind of analysis we did could be used by others in an offensive sense, specifically to conduct the reconnaissance, the first stage of the cyber security kill chain^17^. Albeit many cybercrime gangs currently declared they would not want to hit healthcare services, the current “ransomware” threat model can change in the future, especially if we consider cyber warfare scenarios.

### Mitigation and improvements

There are several possible actions that could be conducted at different levels, to mitigate the presence of vulnerabilities in medical devices. Here we present a discussion of them, based on the results of this analysis:

**Network segmentation and firewalling**: In almost all the analyzed ICS Medical Advisories, CISA recommends users take defensive measures to minimize the risk of exploitation such as restricting system access to authorized personnel only. These recommendations, implemented for example with network segmentation, seem to us to be absolutely essential. Indeed, from this analysis 42% of the attacks allow remote exploitation, and 92% have low complexity. However, this is not always possible for different reasons. One reason is that internet access enhances products and enables product differentiation on the market. For instance, Medfusion 4000 Wireless Syringe Infusion Pump (CVE-2017-12725) uses the internet to update to the drug library and pump software without physically handling the pumps. Another reason is that some of the devices are not designed to be inside a protected healthcare facility. This is the case of Minimed 508 insulin pump (CVE-2018-14781), designed to improve the quality of life of patients by following them as being handheld devices. Finally, with the growing interest around Point-of-care^18^, physical and IT security cannot always be guaranteed. Nonetheless, using appropriate guidelines for network management certainly improves resilience to cyber risk, such as following NIST’s *Securing Wireless Infusion Pumps in Healthcare Delivery Organizations*^19^.

**Improving the efficiency of recalls**: The recall process is a multifaceted operation involving communication from manufacturers to healthcare providers and patients. This notification sequence can be prone to fragility and delays, resulting from challenges in identifying responsible parties for product removal from hospital inventories^10^. Although manufacturers may swiftly pinpoint the majority of devices involved in a recall through returns or destruction notifications, achieving complete identification and tracking of all devices may necessitate months of ongoing communication with providers or distributors. This issue extends beyond cybersecurity to encompass general MD’s safety concerns as well. A noteworthy instance involved the recall of specific mechanical ventilators due to potential health hazards; the recall was initiated two months post-discovery^10^. Despite having elapsed two years, 8957 devices remained unaccounted for according to the manufacturer’s data^20^. Improving the efficiency of the notification chain that starts with Medical Device Reports (MDRs) and goes all the way through to effective problem resolution by recall is certainly something that deserves sensible efforts.

**Acting on regulation**: Acting on regulation is certainly a way to proceed and governments are already moving to force a greater focus on cybersecurity on medical device manufacturers. This includes improvements in the pre-market phase with risk assessment and the post-market phase with cybersecurity surveillance. The most important acts in this regard are the US Consolidated Appropriations Act 2023, and the PATCH Act. In the EU, the Cyber Resilience Act (CRA) is still under development, but it has the potential to have a significant impact on the IoT security landscape. The CRA is expected to require organizations to implement a number of security measures for their IoT devices, such as secure software updates and vulnerability management processes. Organizations should start preparing for the Cyber Resilience Act now by reviewing their IoT security practices and making necessary changes.

**Transparency on software supply-chain**: Government initiatives to keep track of software dependencies will also have a prominent role in prevention and incident response. This is the case of the SBOM (Software Bill Of Material), which device manufacturers must send to the FDA Secretary according to the Consolidated Appropriations Act 2023. Such measures prove particularly valuable when software supply chain vulnerabilities arise, as evidenced by incidents such as CVE-2019-3463 affecting Linux systems and subsequently impacting Valleylab FT10 Energy Platform (ICSMA-19-311-02), or CVE-2017-7269 targeting Microsoft’s Internet Information Server and consequently impacting Siemens Molecular Imaging systems (ICSMA-17-215-01). It is also worth noting that the use of hardened minimal operating systems (e.g., GyroidOS^21^) instead of more generic systems (e.g., Ubuntu) contributes to restricting the attack surface.

**Promoting the use of standards**: The use of standards can mitigate or prevent the rise of some cyber security problems, especially when the origin is accountable to poor product design from the security standpoint. For instance, the CVE-2021-33882 describes a total lack of authentication on proprietary networking commands in an infusion pump^15^. This lack would emerge also by filling the Manufacturers Disclosure Statement for Medical Device Security (specifically question id NAUT-1 of the $MDS^2$ form), but also from the *Application of risk management for IT-networks incorporating medical devices* IEC TR 80001-2-2:2012 (Section 5.11, NAUT) or *Security and Privacy Controls for Information Systems and Organizations* NIST SP 800-53 Rev. 4 (SC-23).

**Raising awareness**: Awareness is an information problem; too much or too broad information clearly impacts the ability to focus on the relevant and important aspects. In this regard, there is a growing need for solutions that provide high-level but targeted information, able to reach also to managerial and non-technical staff. With this work, we want to provide a contribution to this regard. Dedicated tools (e.g., such as active inventory systems) can be an effective instrument in the hands of an authority to increase the cost-benefit of security audits.

**Making use of software security tests**: Finally, the use of security tests (e.g., Google’s OSS fuzzer) or static code analyzers (e.g., Altran’s Code Defect AI) are valid instruments in the hands of manufacturers, agencies, and researchers to improve the quality of the software running on medical devices. We expect that such tools will experience significant improvements given by AI’s advances in handling language models comprising programming languages.

## Methods

### OSINT methodology

Open Source Intelligence (OSINT) is a crucial intelligence technology that utilizes publicly available and accessible information sources to gather insights on stakeholder activities.

The OSINT process^22, 23^ is a comprehensive procedure involving several steps needed to accurately collect, analyze, and process the acquired data. From our technical-scientific perspective, we find the following steps particularly relevant:

1. **Target identification**: This constitutes the initial phase, which establishes the investigation’s objectives and the desired outcomes of the gathered information. Our aim is to concentrate on cybersecurity vulnerabilities in deployed medical devices listed in the EU EUDAMED database or US FDA^24^. For the purpose of this analysis, we only focus on physical devices and not IT infrastructure or component issues (e.g., operating system or virtualization).
2. **Information gathering**: This phase involves searching for and retrieving information from various available data sources. In our case, we collect information from a combination of government data and public cybersecurity databases
3. **Information processing**: During this phase, information is organized and structured for easy comprehension. We processed the data in our case to simplify feature extraction and create external links by defining keywords.
4. **Data analysis**: This stage consists of augmenting data and correlating it with other datasets to interpret and categorize remaining information after prior filtering. Details of this step can also be found in the “Data mining” section.
5. **Dissemination of results**: The culminating step in an OSINT process involves sharing the findings to inform strategic decision-making. As an ongoing process, this stage is partially carried out through our current work.

A practical example of this tool’s application can be found in the article “Open-Source Intelligence for Risk Assessment” by Darren H. Hayes and Francesco Cappa^25^.

### Dataset

To construct the tool, we cross-referenced and manipulated data from numerous datasets from different worlds: cybersecurity databases, government open data, and medical information. We briefly describe the datasets consulted and then how they were used.

#### Government data on contracting

Our analysis began with a review of open data provided by numerous governments related to public administration contracts and made public for the sake of transparency. Much of this data adheres to the Open Contracting Data Standard (OCDS), an international standard for publishing public contract data. The OCDS covers many public and private procurement types, including goods, services, public works, and concessions. Its primary goal is to make public contract data more accessible, allowing for easy comparison and utilization by various stakeholders such as citizens, businesses, organizations, and public authorities. This increased accessibility fosters heightened accountability, improved efficiency, and reduced corruption problems in procurement processes. The OCDS mandates specific fields for publishing public contract data—tender activities, tender participants’ details, payment terms, property rights information, supporting documents—while also offering optional fields. The Open Contracting Partnership^26^, an international non-profit organization committed to promoting transparency and efficiency in worldwide public contracts development was behind the creation of the OCDS.

Data adhering to the OCDS standard can be downloaded from the Open Contracting Partnership’s website for numerous countries across the globe. This data has been utilized, albeit rarely, for research purposes, for instance, anomaly detection in public procurement practices^27^.

In the specific case of the European community, much of this data was generated thanks to the Digiwhist project^28^, a European Union-funded research initiative that uses the OCDS standard to gather and analyze vast quantities of European public contract data, resulting in the creation of a comprehensive European public procurement information database. However, data authored by Digiwhist was found to lack the necessary precision for our purposes, as it generally makes it hard to identify the specific types and models of medical devices procured by public administrations. Consequently, we had to supplement our analysis with additional data provided by national authorities of different countries, including Italy’s Anti-Corruption Authority (ANAC) and open data portals from Romania and Portugal.

#### Cybersecurity information

We used several classification systems for cybersecurity data including the dictionary of Common Vulnerabilities and Exposures (CVEs) and related data from the *National Institute of Standards and Technology* (NIST) National Vulnerability Database (NVD)^29^ regarding platforms, weaknesses, and severity of vulnerabilities. Here is a summary of the main data sources we employed:

- **Common vulnerabilities and exposures (CVE)**: CVE refers to a standardized classification system used for identifying computer vulnerabilities in software and data processing systems. Published by the MITRE Corporation, a non-profit research and technology development organization backed by the Cybersecurity and Infrastructure Security Agency (CISA) under the US Department of Homeland Security (DHS), this list of vulnerabilities is available in a public database on the CVE system’s website. It contains detailed information about each identified vulnerability, with over two hundred thousand CVE records presently listed. The CVE list offers essential information without including technical data, risk details, vulnerability scope, or potential risk mitigation measures.
- **Common platform enumeration (CPE)**: CPE serves as a standard for uniquely identifying and describing hardware and software products. While CVE provides vulnerability descriptions, it does not exhaustively allow for vulnerability detection since this depends on the context in which it occurs, such as the employed hardware, operating system, or software. Consequently, the Common Platform Enumeration standard is used to identify vulnerabilities within specific configurations. Each product is identified by a CPE-ID assigned by an authorized source, which encompasses vendor data, product data, and version. CPE-ID includes, among other information, vendor data, product data and version.
- **Common weakness enumeration (CWE)**: CWE refers to an inventory of common vulnerabilities found in software applications. Developed by the MITRE Corporation with support from the United States Computer Emergency Readiness Team (US-CERT), National Security Agency (NSA), and United States Department of Defense, this classification system is used to categorize weaknesses and vulnerabilities during an application’s development and testing process. This process eventually results in known vulnerabilities that can be identified using CVE. Each CWE code includes a detailed description of the associated vulnerability, related CWEs, potentially risky technologies, and recommended countermeasures to prevent attacks. For example, CWE-89 is the identification number for SQL Injection vulnerabilities and its name is *Improper Neutralisation of Special Elements used in an SQL Command (‘SQL Injection’)*.
- **Common vulnerability scoring system (CVSS)**: CVSS score is a standardized classification system that assesses the risk of a vulnerability in a computer system by assigning it a numerical score from 0 to 10, where 0 represents a minimal risk and 10 a critical risk. The CVSS score is calculated using metrics that consider various aspects of the vulnerability, such as the complexity of the attack, the impact on confidentiality, integrity and system availability, and the existence of mitigation solutions. It consists of three types of scores: basic, temporal, and environmental. The base score, which we utilized in our research, represents the intrinsic and constant features of a vulnerability that do not change over time or across environments.
- **ICSMA**: Additionally, we used the ICS Medical Advisory (ICSMA) by the US Cybersecurity and Infrastructure Security Agency (CISA), which cataloged over 100 medical device issues, providing details on vulnerabilities (CVEs). We combined this dataset with medical device data.

#### Classification of medical devices

We gathered data on medical devices from three sources: the medical device list, risk class, and European Medical Device Nomenclature. These datasets are available under the Medical Device Regulation (MDR), an EU regulation that governs the marketing, distribution, and usage of medical devices within European territories (Regulation (EU) 2017/745). MDR sets safety, quality, and performance standards for CE marking on medical devices sold in the European Union. The regulation includes provisions for creating a European database (EUDAMED) for medical devices and introduces a new classification system based on four levels of risk concerning their medical utility and potential harm to users in case of malfunction or improper use. Specifically, it outlines the following risk classes:

- *Class I*: medical devices with low potential risk to the end user’s health.
- *Class IIa*: medical devices that are moderately invasive and may have an impact on patient health if not used correctly. This class includes devices such as diagnostic imaging systems and electrocardiographs.
- *Class IIb*: medical devices that are more invasive and may have a significant impact on patient health if not used correctly. This class includes devices such as computed tomography (CT) equipment, ultrasound imaging systems and infusion pumps.
- *Class III*: medical devices with a high risk to the health of the user. This class includes all implantable and active devices, such as defibrillators, cardiac aids and joint replacements.

Article 26 of the MDR mandates that the European Commission must offer an internationally recognized medical device nomenclature at no cost. This is supplied through the European Medical Device Nomenclature (EMDN), which manufacturers utilize to register medical devices in the EUDAMED database.

The EMDN features an alphanumeric structure arranged in a seven-tier hierarchical tree. The first level identifies a category (e.g. dialysis device), the second level indicates the group (e.g. dialysis filters), and the third represents various types, which may extend into multiple levels of detail.

### Data mining

**Figure 5:**
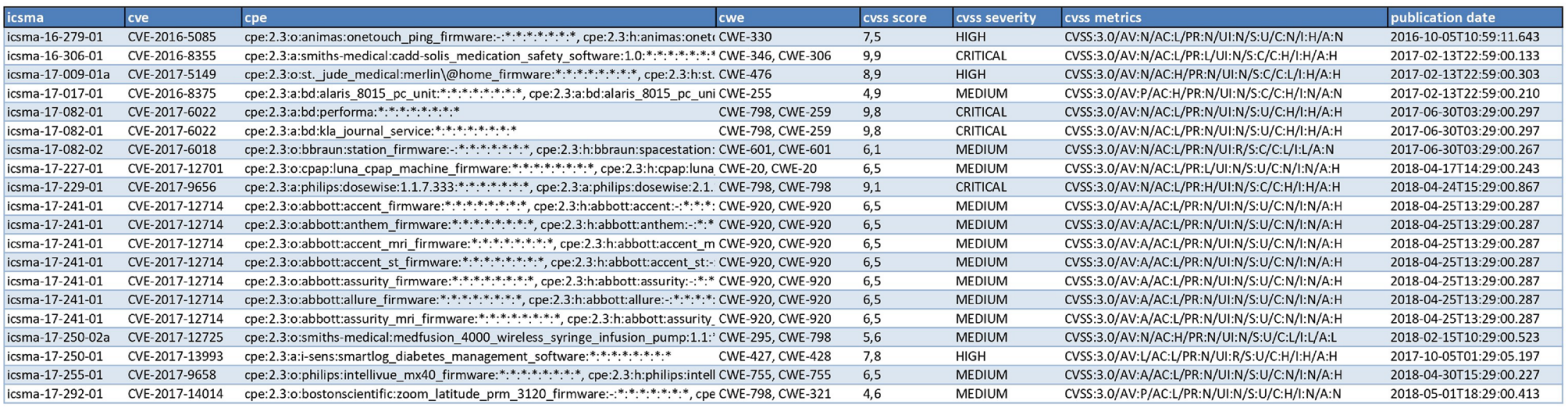
Extract of the file containing ICSMA notices and associated CVE, CPE and CWE.

We initiated our analysis with ICSMA data, extrapolating with a crawler the medical device vulnerabilities (CVS). We then enriched this information by linking CWE, CPE, and CVSS datasets for each vulnerability, as illustrated in Fig. 5. We manually analyzed the data to focus on relevant vulnerabilities, eliminating those related to operating systems or virtual machines, and maintaining only those specific to recognized medical devices.

Then, we transitioned from identifying CPEs to crafting relevant regular expressions capturing specific models of medical devices. We utilized these expressions to search through OCDS files’ description fields and national open data. Our analysis spanned several days, which were needed to process the 92 million textual descriptions that encompassed purchasing histories.

**Figure 6:**
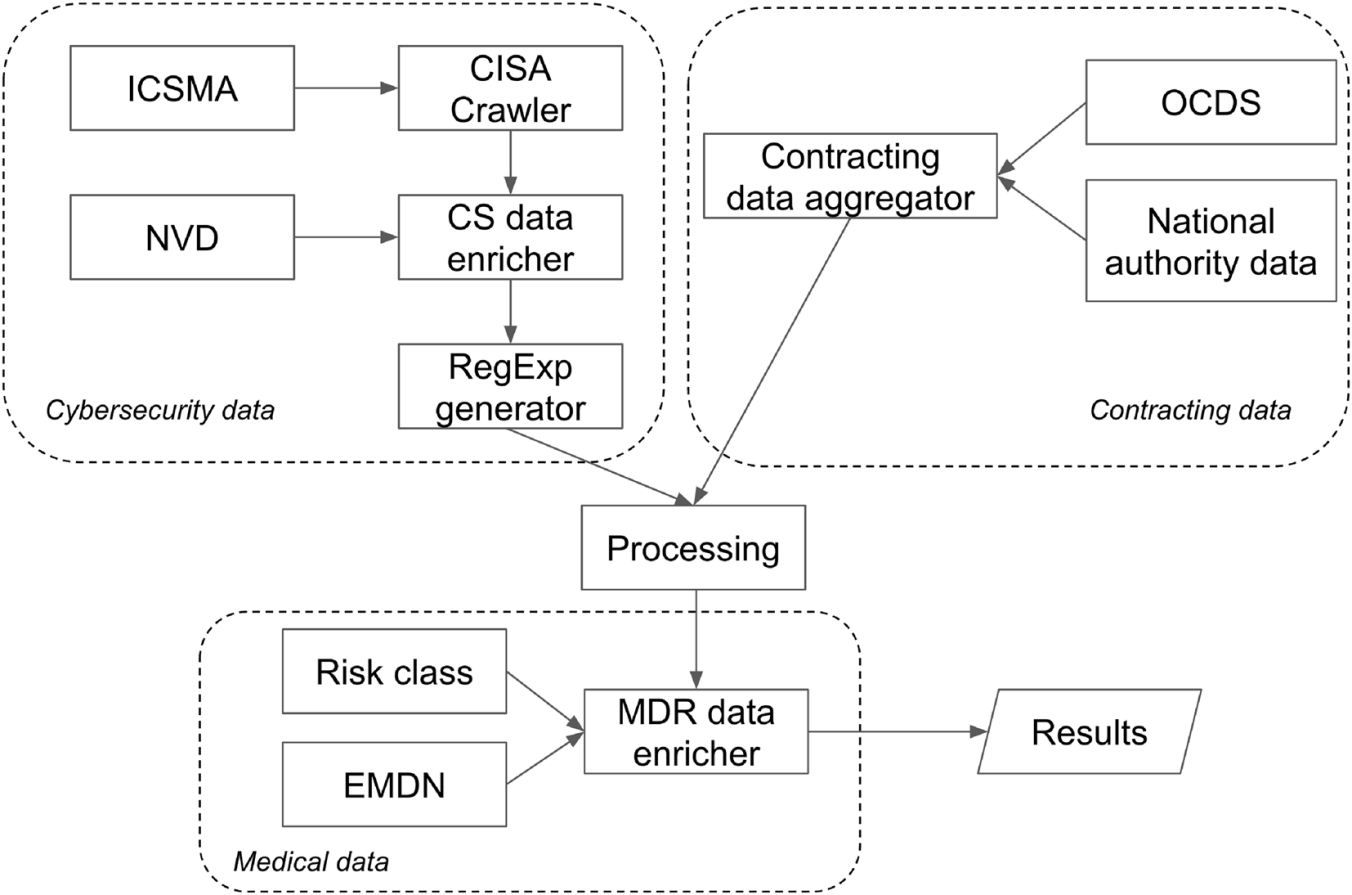
Block diagram of the applied data mining technique.

We examined and enriched the acquired data by incorporating ECDN cataloging and truncating it at the third level for aggregation purposes. Lastly, we recorded each medical device’s associated risk class. Figure 6 shows a block diagram of our methodology.

### Ethics and lawfulness of the analysis

The detailed results of the analysis are not disclosed due to ethical considerations, as they can be utilized to create a comprehensive map for executing the initial phase of a kill chain^17^. All utilized datasets were made available as open data specifically for this purpose. Precisely, CVE, CWE, CPE data are published to examine vulnerabilities and provide a unique identification for them; medical device risk data are disclosed for transparency by the European Union and FDA to offer visibility into risk; OCDS data are made public to facilitate transparency and oversight over public acquisitions and to enable audits, such as the one conducted within this study.

### Limitation of the analysis and extension

The methodology proposed in this paper has the strong advantage of being based on open data, thus not requiring interactivity with healthcare facilities. It has inherent limitations, however, due largely to the analysis of free text strings that are the contract descriptions. In the search phase, it is possible to have false negatives, for example, if the description does not include the make and model of the medical device purchased (or if it is miswritten), or, in rarer cases, false positives that have been minimized through manual analysis and an iterative development cycle.

Then, each match found does not necessarily correspond to a vulnerable device today. First, it is possible that the device has been patched. Second, matches may also deliberately refer to consumables (e.g. defibrillator pads) of a device that had a vulnerability. Looking at the data, we found out that in some cases it would be possible to annotate the number of devices purchased in a contract. This type of analysis requires more sophisticated techniques (e.g., Named Entity Recognition) and is a possible future development of this work.

Finally, we remark that many MDs have many software dependencies (libraries, operating systems etc.). For instance, MRI computers typically run common operating systems such as Microsoft Windows. As the FDA emphasized in their “Ensuring Cybersecurity of Devices” section, having a detailed “bill of materials” is essential^8^. Access to this information would enable us to adopt successful strategies from the software industry, like utilizing bots that link security issues with software project dependencies^30^.

### Related work

#### Regulation

In the United States, the Consolidated Appropriations Act of 2023 (“Omnibus”) has mandated the Food and Drug Administration (FDA) to incorporate cybersecurity as a crucial aspect in their evaluation of specific medical devices containing software with internet connectivity capabilities. A “cyber device” is defined as an apparatus that comprises software that has been validated, instated, or authorized by the sponsor for use either within or as a device, possesses internet connectivity capabilities, and includes technological characteristics that have been validated, instated, or authorized by the sponsor, rendering them susceptible to cybersecurity threats. As of March 29, 2023, individuals submitting premarket applications for devices meeting the criteria of a cyber device must also include a plan to monitor, identify, and remediate postmarket cybersecurity vulnerabilities and exploits. This plan necessitates coordinated vulnerability disclosure and corresponding procedures to maintain continual post-market cybersecurity surveillance. Furthermore, cyber device manufacturers are obliged to supply the FDA with a comprehensive “software bill of materials” (SBOM)—delineating all commercial, open-source, and off-the-shelf software utilized in medical devices.

The Protecting and Transforming Cyber Health Care (PATCH) Act was introduced to the United States Congress in March 2022. This legislation aims to bolster cybersecurity requirements for medical devices further and mandates healthcare organizations to establish cybersecurity measures safeguarding their medical devices.

For the European Union, the primary regulations concerning medical devices consist of 745/2017 (MDR) and 746/2017 (IVDR), which were adopted and enforced on May 25th, 2017.

Annex I of the Medical Devices Regulations outlines cybersecurity requirements addressing both premarket and post-market considerations. Key points, summarized in^12^, include the implementation of risk control measures, a comprehensive risk management throughout the device’s lifecycle, and the establishment of minimal IT security requirements.

#### Standards

There is a large variety of standards that can be applied for the development and design of the risk assessment of medical devices. In^31, 32^, authors report part of such standards. As they noted, although many standards do not cope directly with cybersecurity, they may cover design aspects that can be related, since many vulnerabilities arise from poor software design.

It is also important to point out the presence of additional and optional important documentation, such as the Manufacturers Disclosure Statement for Medical Device Security that consists of a form (MDS$^2$ form ) with true/false answers, focussing on roles and responsibilities.

The questions have counterparts in standard frameworks dealing with risk management, such as IEC TR 80001-2-2:2012 NIST SP 800-53 Rev. 4 ISO 27002:2013. The form is endorsed by the American College of Clinical Engineering (ACCE), the ECRI (formerly the Emergency Care Research Institute), the Healthcare Information and Management Systems Society (HIMSS), and the National Electrical Manufacturers Association (NEMA). This additional documentation can be used by customers of medical device firms to guide their choice as it improves the transparency and the evaluation of cybersecurity. However, it clearly has the limitations of any true/false questionnaire: it cannot cover the many details in which vulnerabilities may lurk. For example, it asks if there is encryption for the data at rest, but it is well known that also improper use of encryption may bring vulnerabilities such as the famous Zerologon (CVE-2020-1472).

Finally, the many IoT cybersecurity standards, such as the NIST’s 8259A “IoT Device Cybersecurity Capability Core Baseline”^33^ and ETSI 103.645 “CYBER; Cybersecurity assessment for consumer IoT products” contain important guidelines that can help the construction of more secure medical devices dealing with device identification, authorized configuration, data protection, restricted access or software updates.

#### Analysis of cybersecurity for medical devices

The complexity of cybersecurity in medical devices has been addressed in various ways by experts in the field. Numerous studies focus on identifying and understanding the risks associated with attacks on medical devices, including the examination of national and international legal documents, policy reports, industry frameworks, cyber breach analyses, and academic journal articles^31, 34, 35^. These works assess the effectiveness of current policy measures in securing the Internet of Medical Things technologies.

Reports such as the “2023 State of Cybersecurity for Medical Devices and Healthcare Systems”^36^ by Health-ISAC, Finite State, and Securin outline vulnerabilities related to medical devices, software applications, and healthcare systems. Their research discovered 993 vulnerabilities within 966 medical products and devices, revealing a 59% increase from 2022. The majority of these vulnerabilities, 64%, were found in software, while 16% have been weaponized.

A number of researchers utilize open data to conduct their analyses, similar to this study. For instance, Stern et al.^37^ analyzed data from publicly accessible FDA product summaries and found that 13.79% contained software elements while only 2.13% contained cybersecurity-related information within the summaries. Additionally, there are studies that show how a large proportion of recall procedures are ascribable to software problems^9, 38^.

To the best of our knowledge, there have been no large-scale analyses of vulnerabilities related to medical devices in use in healthcare facilities like the one lent in this paper. There are, however, several specific analyses that relate to the spread and impact of a single vulnerability^35^. The OCDS data used in this paper also have been little used in the literature and mostly for identification of anomalies in public procurement practices^27^, never for cybersecurity.

## Data Availability

The datasets used and/or analyzed during the current study are available from the corresponding author upon reasonable request.
